# A universal preference for animate agents in hominids

**DOI:** 10.1016/j.isci.2024.109996

**Published:** 2024-05-16

**Authors:** Sarah Brocard, Vanessa A.D. Wilson, Chloé Berton, Klaus Zuberbühler, Balthasar Bickel

**Affiliations:** 1 Department of Comparative Cognition, Institute of Biology, University of Neuchatel, Neuchatel, Switzerland; 2 Department of Comparative Language Science, University of Zurich, Zurich, Switzerland; 3 Center for the Interdisciplinary Study of Language Evolution (ISLE), University of Zurich, Zurich, Switzerland; 4 School of Psychology & Neuroscience, University of St Andrews, St Andrews, Scotland (UK)

**Keywords:** biological sciences, evolutionary biology, natural language processing, neuroscience

## Abstract

When conversing, humans instantaneously predict meaning from fragmentary and ambiguous mspeech, long before utterance completion. They do this by integrating priors (initial assumptions about the world) with contextual evidence to rapidly decide on the most likely meaning. One powerful prior is attentional preference for agents, which biases sentence processing but universally so only if agents are animate. Here, we investigate the evolutionary origins of this preference, by allowing chimpanzees, gorillas, orangutans, human children, and adults to freely choose between agents and patients in still images, following video clips depicting their dyadic interaction. All participants preferred animate (and occasionally inanimate) agents, although the effect was attenuated if patients were also animate. The findings suggest that a preference for animate agents evolved before language and is not reducible to simple perceptual biases. To conclude, both humans and great apes prefer animate agents in decision tasks, echoing a universal prior in human language processing.

## Highlights

• All species tested showed a robust preference for animate agents acting on objects
• The preference sometimes generalized to inanimate agents but never to animate patients
• The preference is specific to decision task and does not generalize to event perception
• The preference parallels the agent preference guiding sentence processing in humans

## Graphical abstract

**Figure:**
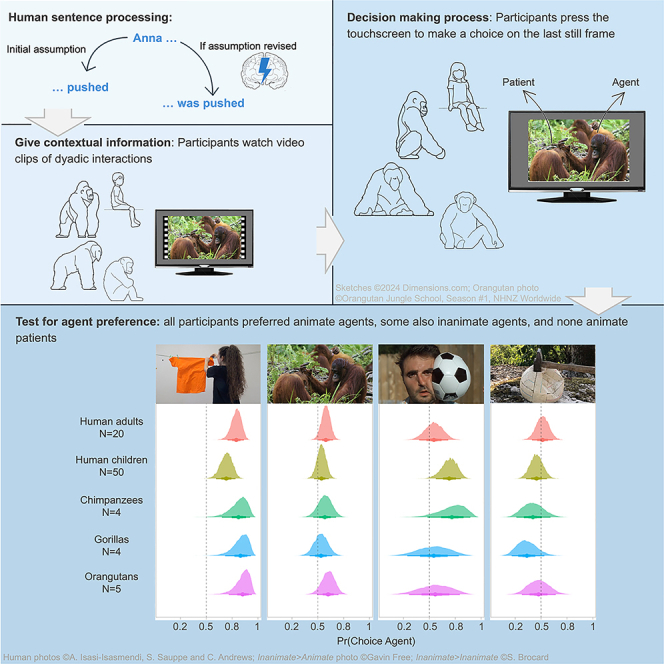

## Introduction

Understanding spoken language is a mostly effortless and instantaneous process, despite the fact that speech tends to be rapid and information-rich, but often also fragmented and uncertain. Listeners make on-the-fly decisions about meanings as each word is received long before, and regardless of, utterance completion.^1^^,^^2^^,^^3^ These real-time decisions critically rely on integrating current linguistic (e.g., lexical) and non-linguistic (e.g., contextual) sensory information^4^ with prior expectations about intended meanings,^5^^,^^6^ i.e., beliefs about plausible outcomes before evidence is observed.^7^^,^^8^

One powerful prior in humans is the disposition to preferentially assume that any person or animate being first mentioned in a sentence will play an agent (rather than a patient) role in an upcoming interaction — in other words, that they will instigate rather than undergo an event. Specifically, sentence processing is guided across languages by a prior expectation that role-ambiguous noun phrases default to an agent interpretation. When this expectation fails upon utterance completion, it triggers electrophysiologically measurable self-correction.^9^^,^^10^^,^^11^^,^^12^^,^^13^ Language models that include this agent preference outperform simpler models in predicting event-related potentials (chiefly, the N400 effect as an indicator of role prediction failure) across structurally highly diverse languages.^14^ This prior is universally robust, particularly for animate agents. The preference can be overridden for inanimate agents (as in “the waves smashed the canoes” or “this water makes me sick”) but, as far as is currently known, only in the very few languages that place agents last in sentences.^15^ The prior furthermore leads to simpler, “unmarked” encoding of animate agents across languages,^13^^,^^16^^,^^17^^,^^18^ although this often results in increased neural activity during sentence planning due to uncertainty.^19^^,^^20^ The agent preference in language processing is further echoed by an attentional preference for agents when looking at still images,^21^^,^^22^^,^^23^^,^^24^^,^^25^ even when they are presented only as briefly as the blink of an eye, demanding a high-speed decision.^26^^,^^27^ This suggests that event roles can even be rapidly extracted from still images when participants engage in appropriate tasks.

The origin of the agent preference in language processing remains unclear. One hypothesis that derives from the above literature is that it is a by-product of linguistic communication, mirroring a universal trend across languages toward expressing agents before patients^28^^,^^29^^,^^30^^,^^31^^,^^32^^,^^33^ as the prime topics of conversational interest. Only very few languages place agents last in sentences, such as Äiwoo spoken on the Solomon Islands.^15^ The overwhelming majority of languages exhibit a preference for placing the agent first, and this tendency extends to subjects requested to communicate about an event with gestures instead of words.^29^^,^^31^ A possible driver of this ordering preference is that agents are more commonly maintained as the topic of interest in conversations^34^^,^^35^ and are therefore easier to retrieve during speech planning than patients, leading to earlier production.^36^^,^^37^^,^^38^ However, recent work suggests that these order preferences might be primarily driven by a generally stronger interest in human and animate referents than in inanimate referents, independent of their roles.^39^^,^^40^

An alternative hypothesis is that the agent preference is born out of more basic non-verbal core cognition,^41^ based on the assumption that early recognition of agents is evolutionarily important for understanding and predicting events, such as social interactions between competing group members or predicting predator behavior. This hypothesis is consistent with the fact that the human brain has a tendency to assign causality to almost any interaction, independent of language,^42^ and that even pre-verbal children assign agent and patient roles to entities of events.^41^ The preference of agents might therefore have evolved long before the emergence of language,^43^ possibly as a mechanism to predict the outcome of events in a more general attempt to reduce uncertainty.^7^ However, such an agent preference might be more nuanced than initially presumed.^43^ It may not generalize to any agent (i.e., animate or inanimate), as human preference for agent can vary according to the context, and appears fully resilient only when agents are animate.^15^

Here, we investigated the universal human preference for agents with a comparative study on our closest living relatives—four western chimpanzees (*Pan troglodytes verus*), four western lowland gorillas (*Gorilla gorilla gorilla*), and five Sumatran orangutans (*Pongo abelii*)—and 20 human adults and 50 children of different ages. If the agent preference was pre-linguistic, we expected it to be shared across apes; if it is a by-product of usage patterns in language, we expected it to be specific to humans, possibly subject to developmental patterns.

Assessing agent preference non-linguistically is a challenging task since it predominantly emerges during sentence processing or picture viewing. Here, we developed an ape-friendly, non-linguistic approach that recreates the decision processes when contextual information is integrated with prior expectations. First, participants received contextual information by watching short video clips of natural events to mimic the continuous flow of incoming information in language. We used natural scenes with different hominid species to promote socio-ecological validity of the stimuli. For this reason, we did not standardize the videos in favor of reflecting the diversity of events encountered in the natural world. Then, with customized touchscreen devices,^44^ we tested the decision-making process to access any eventual preference.

We used simple, unrestricted setups that allowed similar testing conditions for all participants, and the protocol was engaging to enhance participation from both great apes and children from 2.5 to 12 years old. Prior to the test, great apes underwent a three-stage training process that involved watching video clips featuring a single unknown conspecific actor (i.e., without event roles), while human volunteers received instructions but remained naive to the goal of the experiment (more details in the STAR methods). Participants then watched videos of two entities naturally interacting in clear causal *agent>patient* relations (“>” stands for “acts on”). At the end of the video, the last still frame remained on screen and participants had to make a choice by touching either the agent or the patient, in the absence of any additional information and without any subsequent reward (Figure S1).

Events were divided into four conditions: (1) *Animate>Inanimate*: an animate agent (chimpanzee, gorilla, orangutan, human or non-primate) acting on an inanimate patient (e.g., a cow playing with a ball, an orangutan manipulating Lego blocks), (2) *Animate>Animate*: an animate agent acting on a conspecific in different types of interactions, i.e., agonistic (e.g., aggressions), play, affiliative (e.g., grooming), or cooperative (e.g., help), (3) *Inanimate>Animate*: an inanimate agent (i.e., object) acting on a human patient (e.g., a ball smashing into the face of a person), and (4) *Inanimate>Inanimate*: an inanimate agent (different objects, such as balls, cars, and trees) acting on another object in different types of interaction (e.g., colliding, falling on; see STAR methods).

## Results

We first tested whether the event role distinction (agent vs. patient) was predictive of decisions, above and beyond relevant covariates (side of choice, conditions, species of the participant, size of the actors, and type of stimuli; see Table S1). We found that a Bayesian log-linear model with event role distinction had much better predictive performance than a model without these role distinctions, under leave-one-out cross-validation (elpd_*Full model*_ = −703.3, SE = 12.6 and elpd_*Model without event roles*_ = −4040.2, SE = 290.7; see STAR methods for detailed explanations).

We then estimated agent choice directly in a multi-level Bayesian logistic model, retaining only covariates that were needed to maintain predictive performance under leave-one-out cross-validation (elpd_*Full model*_ = −4399.5, SE = 32.2 and elpd_*Simplified model*_ = −4399.4, SE = 31.5): condition (e.g., *Animate>Animate*, *Animate>Inanimate*), species of the participant, side of choice, side of agent, status of event (i.e., action still on-going or completed), self-propelledness of agent (i.e., agent moves without propulsion or agency), centrality of agent (i.e., closeness of agent to the center of the screen relative to the patient), and differences in movement between agent and patient (i.e., difference in total amount of time agent and patient moved during the event) (Figure S2; see Table S1 for details on the covariates).

Our model revealed a strong effect of the condition (see Tables S2‒S4 for posterior estimates), after controlling for the retained covariates. We declared a preference in agent choice as “robust” if at least 90% of the posterior estimates were above chance, i.e., a 0.5 probability of agent choice.^45^

**Figure 1:**
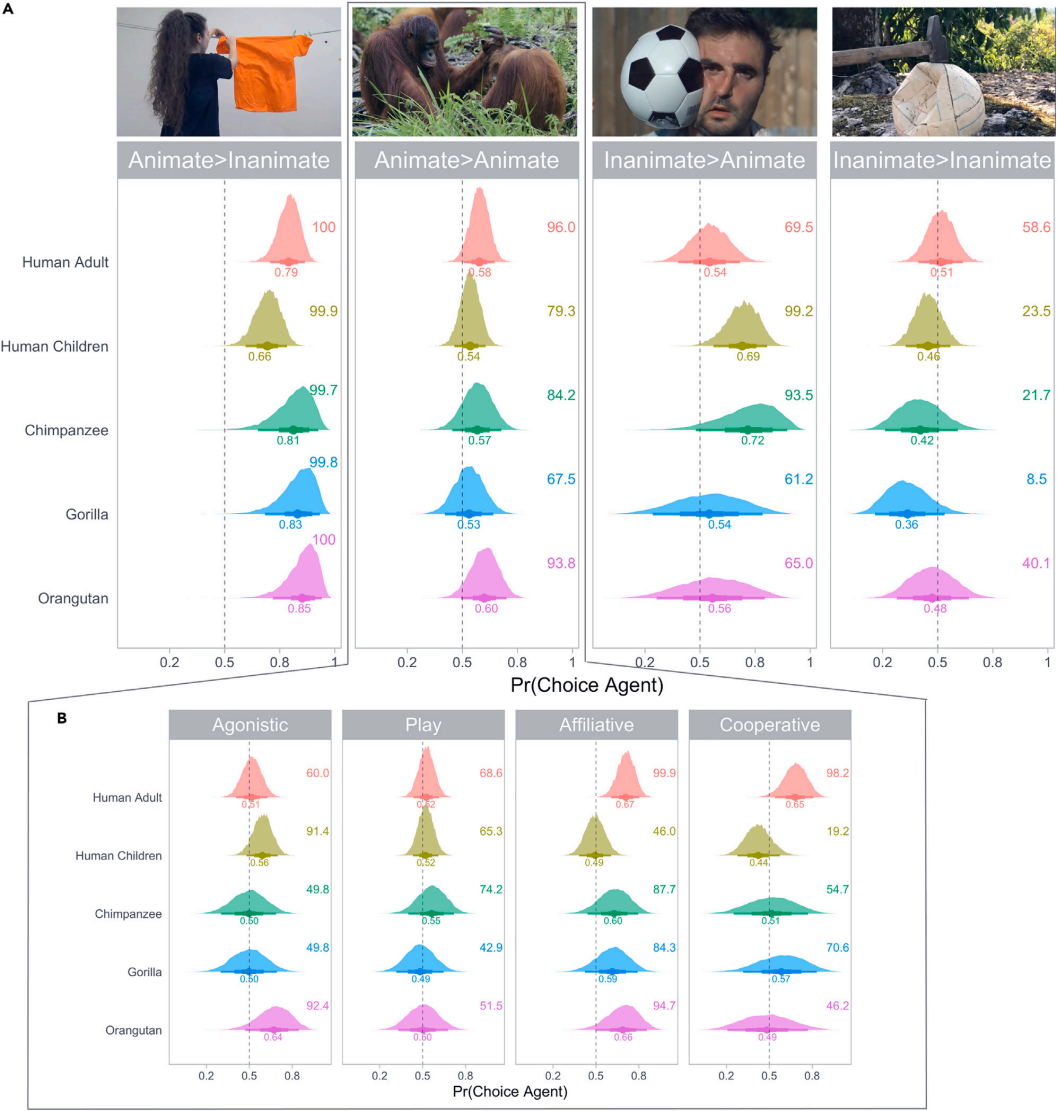
Posterior probability of the agent choice for each species across (A) Across conditions and (B) across type of interaction. The dashed line corresponds to a random choice (0.5). The points represent the mean of the estimates and the three levels of thickness of the point intervals represent the 30%, 60%, and 90% credible intervals. Values presented next to each distribution are the percentages of posterior estimates above a 0.5 probability of agent choice. Twenty human adults, 50 children, four chimpanzees, four gorillas, and five orangutans were tested for each condition, but one adult was removed from the *Animate>Animate* condition (“>” stands for “acting on”), as she was identified as an outlier based on her age. Twenty-five clips were presented in the *Animate>Inanimate* condition, 74 in the *Animate>Animate* condition (19 clips depicted an agonistic interaction, 27, 20, and 7 clips represented play, affiliative, and cooperative interactions, respectively), 15 in the *Inanimate>Animate* condition, and 41 in the *Inanimate>Inanimate* condition. See also Figure S3 and Tables S2‒S5. Photo credits: (*Animate>Inanimate*) A. Isasi-Isasmendi, S. Sauppe, and C. Andrews; (*Animate>Animate*) Orangutan Jungle School, Season #1, NHNZ Worldwide; (*Inanimate>Animate*) GAVIN FREE and (*Inanimate>Inanimate*) S. Brocard.

In the *Animate>Inanimate* condition, we found that all species had a robust preference for the agent (all Pr(choice>0.5) > 0.99; Figure 1A, first panel). In humans, we found an age effect insofar as agent preference increased with age, most steeply for young adults (Figure S3; Table S5). For the apes, estimates were uncertain because of the small number of subjects.

In the *Animate>Animate* condition, arguably much more complex, the agent preference was robust only in adult humans and orangutans (Figure 1A, second panel, Pr(choice>0.5) > 0.93) with no age effect in humans (Figure S3; Table S5). Closer inspection of the specific content suggested that the effects were slightly different in human adults and orangutans (Figure 1B). While both showed a preference in affiliative settings (Pr(choice>0.5) > 0.94), humans additionally showed it in cooperative settings (Pr(choice>0.5) > 0.98) while orangutans showed it in agonistic settings (Pr(choice>0.5) > 0.92). Apart from these, we found an agent preference only in human children in the agonistic setting (Pr(choice>0.5) > 0.91), similar to the orangutans. In all other settings, the *Animate>Animate* condition elicited a balanced preference to agents and patients.

Since the first two conditions did not allow to disentangle the effect of animacy from agency and the agent preference was universally robust only in the *Animate>Inanimate* condition, we furthermore tested whether this effect might be driven by animacy differences rather than agency. To do so, we tested another set of videos, this time depicting events involving inanimate agents. In the *Inanimate>Animate* condition, human children and chimpanzees continued to prefer (inanimate) agents over animate patients (Pr(choice>0.5) > 0.93), while for the other species the agent preference weakened considerably compared to the *Animate>Inanimate* condition (Figure 1A, third panel; Figure S3; Table S5). Critically, no participant favored the animate patient, suggesting that the agent preference in the *Animate>Inanimate* condition was driven by a preference for agents and not by a preference for animate entities independent of role.

For inanimate agents acting on inanimate patients (*Inanimate>Inanimate* condition), finally, all species lost their agent preference and, if anything, shifted toward a patient preference (Figure 1A, fourth panel). This reversal was most pronounced in gorillas (posterior mean of patient choice = 0.64, Pr(patient choice>0.5) = 0.91).

## Discussion

We replicated with video stimuli the effect of a powerful prior preference^9^^,^^10^^,^^11^^,^^12^^,^^13^^,^^21^^,^^22^^,^^23^^,^^24^^,^^25^ that in humans is known to guide sentence processing and still picture apprehension: a preference for interpreting an incoming stimulus as representing an agent. In language, this preference has been shown to be universally robust for animate agents, while it can disappear for inanimate agents when the statistical distributions of a language disfavor the agent, notably when they follow a patient-verb-agent word order.^15^

Analogous to this, we found robust preferences for animate agents in all four tested species of hominids. The preference was reduced in the *Animate>Animate* condition, i.e., when the animate agents acted on animate instead of inanimate patients. One possible explanation for this is that video stimuli were perceived as much more complex when they involved two animate entities. If one is animate and the other inanimate, agency is much more straightforward to estimate since animates are more common and more natural agents than inanimates.^46^ Moreover, in events with two animates, there are many additional aspects of interest to viewers, such as age/sex class (male vs. female, adult vs. juvenile), kinship relations, or facial expressions, and these might confound the choice between agent and patient. There is also little doubt that both human and non-human primates are very sensitive to the social factors in this, particularly along the competitive-cooperative divide.^47^^,^^48^ Consistent with this, languages tend to evolve extra case marking when patients are animate,^49^ reflecting the additional complexity of the situation,^17^ although it is debated how universal this trend is.^13^

Another possible explanation of the results lies in the nature of the events themselves. Across species, the agent preference was reduced in events that were intrinsically more symmetrical and reversible, such as reciprocal events in play (e.g., “they chased each other”) or affiliative behavior (e.g., grooming in turns). When participants arrived at the freeze frame, they might already be well aware of the agent/patient roles, but were more interested in the patient, expecting reversal or reciprocity in these types of events. The agent preference was also reduced in agonistic events, arguably because there the patient was of heightened interest (e.g., “he attacked her”) and participants tracked the social consequences (will she fight back? suffer?). Remarkably, however, the agent preference did persist in agonistic contexts in children and orangutans, and it uniquely persisted in humans for cooperative events. This variation calls for targeted follow-up experiments on the role of empathy and cooperation in event cognition across species.

Surprisingly, and contrary to human adults, the agent preference also persisted in the *Inanimate>Animate* condition for human children and chimpanzees. It is notable that chimpanzees, as a species, are much more manipulative and tool-obsessed than other great apes (especially for extractive-foraging and complex tools^50^). In the wild, chimpanzees use tools to impact on the animate world (e.g., crushing of parasites with leaves,^51^ hunting with tools,^52^^,^^53^ and attacking leopards with sticks^54^^,^^55^), which may explain their heightened interest for how objects impact on animate patients. Arguably, the same is true for children, who are in an ontogenetic phase where they still need to learn much about how to navigate in a world of artifacts supplied by their culture. Overall, these findings in the *Inanimate>Animate* condition support the idea that great apes, including humans, do not simply respond to animacy but appear to process something about the causes and consequences relating to the participants of interactions.

Finally, what explains the lack of preference in the *Inanimate>Inanimate* events across species? Of all conditions, these event types were probably perceived as the most unnatural. Moreover, in most cases, the inanimate agent was most likely set in motion by a hidden animate agent (e.g., a ball thrown by *someone*), thus participants had incomplete information and might have struggled to reconcile the perceptual features of the (agent) object with fundamental features of an agent, such as self-propelledness and goal-directedness.^18^^,^^56^ Alternatively, they might have imagined the impact of the invisible agent, with further complicated response patterns.

What was responsible for the universal preference for animate agents? One possibility is that it was chiefly driven by the *Animate>Inanimate* condition, the only condition in which the preference was fully robust for all participants. This condition might reveal a general template that was occasionally extended to events in the *Animate>Animate* condition, likening the animate patient to an inanimate entity because of its reduced activity. Animates indeed were perceptually more salient than inanimates. For example, changes affecting animate elements in complex natural scenes were detected faster and more accurately by human subjects than changes affecting inanimate elements.^57^ In line with this is the fact that, in the *Inanimate>Inanimate* condition, the agent preference disappeared in all species (Figure 1A, fourth panel). However, if animacy were the sole factor guiding choice, subjects should have shown a clear preference for the patient in the *Inanimate>Animate* condition, which was not the case. In addition, this account would predict that the agent preference in the *Animate>Animate* condition would be reduced most strongly in agonistic events, where the patient is least active. Yet the largest reduction was observed in play events, where both activities were more balanced.

Another possibility is that the preference for agents (albeit moderated by various factors) was due to a phylogenetically old but general bias to perceive events in terms of causal forces,^42^ preferring for example, self-propelled entities in abstract visual displays (as shown, e.g., for newly hatched chickens^56^). Under this account, one would expect the preference to extend to all inanimate agents, but this is not what we found. Moreover, agent preference seems specific to decision-making, while sensitivity to causal forces holds for any perceptual processing. For example, in an ongoing companion study using eye-tracking, we found that great apes and humans, watching how events unfold over time, alternated their gaze between the agents and patients, suggesting they were trying to understand the causal nature of the interaction.^58^ In the absence of a request for decision, this gaze alternation did not show a general bias for agents.^58^ Similarly, the diversity of agents encountered across events makes it unlikely that participants relied on a set of complex cues (such as face recognition, facial expression, or position) to make their choices. In addition, such cues would arguably separate animate and inanimate entities more than agents and patients; yet we found no evidence that choices were reducible to preferences for animates or inanimates.

Importantly, our experiment was based on a free-choice paradigm with no specific training or instructions that could have biased preferences toward the agents or patients of events, in any of the species we tested. The fact that participants, across species, still showed consistent degrees of agent preference indicates the presence of a shared, evolutionarily old cognitive predisposition that predates language.

### Limitations of the study

A frequent argument in studies such as this one is that, in the absence of detailed instruction, human participants try to guess the aim of the task, which may affect the nature of their choices. Additionally, apes may carry over biases from previous experiments.^59^ Our ape participants however were fairly new to touchscreen studies and certainly never had experienced anything akin to this study. If anything, any bias would have obstructed the emergence of the observed patterns, rather than produced them.

### Conclusions

Our experiments reveal a universal preference for animate agents across the tested hominids. The preference seems specific to the kind of decision-making that our task required and that also underlies the agent preference in human sentence processing and still picture apprehension. This suggests that the agent preference is a specific mechanism combining agency with animacy. It is conceivable that this mechanism has been selected for in highly encephalized species, due to its enhancement effect on rapid decision-making in event cognition, allowing individuals to quickly recognize and predict the potential impact that an animate entity can have on the perceiver, e.g., as a predator or as a competitor. Our data are consistent with the interpretation that this cognitive prior has evolved considerably earlier than modern humans. Whether or not the same or similar preferences for animate agents are present in other groups of animals is a topic for further research.

## STAR★Methods

### Key resources table

**Table:** 

REAGENT or RESOURCE	SOURCE	IDENTIFIER
**Deposited data**		

Dataset	This paper	https://osf.io/5vrc7/?view_only=3519ecd7e366403d9f8e9e52cb17eb71
Data analyses	This paper	https://osf.io/5vrc7/?view_only=3519ecd7e366403d9f8e9e52cb17eb71

**Software and algorithms**		

MATLAB (version R2017a)	The MathWorks Inc., 2017^60^	https://www.mathworks.com
R (version 4.0.5)	R Core Team, 2021^61^	https://www.r-project.org
Stan	Carpenter et al., 2017^62^	https://doi.org/10.18637/jss.v076.i01

**Other**		

Video stimuli	This paper	https://osf.io/5vrc7/?view_only=3519ecd7e366403d9f8e9e52cb17eb71
Formal definition of the statistical models	This paper	https://osf.io/5vrc7/?view_only=3519ecd7e366403d9f8e9e52cb17eb71

### Resources availability

#### Lead contact

Further information and requests for resources should be directed to and will be fulfilled by the lead contact: Sarah Brocard (sarah.brocard1@gmail.com).

#### Material availability

This study did not generate reagents.

#### Data and code availability

• Data have been deposited at https://osf.io/5vrc7/?view_only=3519ecd7e366403d9f8e9e52cb17eb71 and are publicly available as of the date of publication.
• All original code, analyses and stimuli have been deposited at https://osf.io/5vrc7/?view_only=3519ecd7e366403d9f8e9e52cb17eb71 and is publicly available as of the date of publication. All custom MATLAB scripts will be available upon request.
• Any additional information required to reanalyze the data reported in this paper is available from the lead contact upon request.

### Experimental model and study participant details

#### Ethic statement

This study received approval from the ethics committee of the University of Neuchâtel (project 66-2020-B), the Basel cantonal veterinary office (permit 3077), and the Animal Welfare Officer at Basel Zoo.

#### Participants and study site

##### Humans

We tested two age groups: *(i)* adults (*Animate>Animate* condition: *N* = 19; 11 females; mean 29.95 ± 12.04 years old (an extra participant was removed as she was considered as an age outlier); range [19; 56]; other conditions*: N* = 20; 13 females; mean 24.55 ± 7.88 years old; range [18; 56]), and *(ii)* children from 2 to 12 years old (*Animate>Animate* condition: *N* = 50; 28 females; mean 5.64 ± 2.03 years old; range [2; 11]; other conditions: *N* = 50; 23 females; mean 5.84 ± 2.26 years old; range [2; 12]).

The children and some of the adults were recruited from visitors of the primate house at Basel Zoo (Switzerland), through the zoo’s social media advertisements, posters in the zoo or direct approach. Other adult participants were recruited from amongst students at the University of Neuchâtel via email. All participants were Caucasian.

Prior to the experiment, all volunteer participants were given basic information about the procedure and policies, without knowing the goal of the experiment. They were instructed to watch the short videos and choose one actor by touching the screen when the image paused. Participants were also informed that there was no right or wrong answer. By the end of the experiment, all participants or their legal guardians received a detailed information sheet (background, procedure, confidentiality policy) and had to sign an informed consent form and respond to a short demographic questionnaire. Children also received a participation “certificate”.

##### Great apes

Participants were chimpanzees, gorillas and orangutans housed at Basel Zoo. During the study period, the zoo housed up to 15 chimpanzees, eight gorillas and nine orangutans. Chimpanzees and gorillas were always maintained as respective cohesive social groups and orangutans were kept as adult pairs with their immature offspring. Four chimpanzees (two females; mean 7.25 ± 7.18 years old; range [3; 18]), four gorillas (three females; mean 13.75 ± 12.26 years old; range [5; 31]) and five orangutans (three females; mean 15.40 ± 5.08 years old; range [8; 20]) voluntarily participated in the study (Table S6). All species had access to indoor and outdoor enclosures (total holding area for chimpanzees: 767.43 m^2^, 4,957.03 m^3^; gorillas: 753.22 m^2^, 4,173.89 m^3^; orangutans: 813.81 m^2^, 1,0691.79 m^3^). All enclosures were equipped with ropes, hammocks and climbing structures; material to build nests was also provided every day. The roof of all enclosures was made of sliding glass windows assuring natural lightning throughout the day. All species were fed a mix of fruit and vegetables supplemented with small amounts of protein, with several “meals” distributed throughout the day. The study did not pose any risk and participation was fully voluntary. Subjects were never food or water deprived at any point, but received food rewards for participating, in accordance with their diet.

### Methods details

#### General procedure

Training of the great apes and testing data were collected every weekday from 8:00 to 11:00 for the great apes and from 9:00 to 16:00 for human participants. We used touchscreen devices (Iiyama ProLite T1931SR, 19″, 48 cm and 1280 × 1024 resolution, using resistive technology) connected to a laptop (Dell Latitude 5580). The experiments were programmed and displayed using MATLAB software (The MathWorks Inc. (2022), version R2017a) and specifically the Psychophysics Toolbox Version 3 (PTB-3) extensions.^63^^,^^64^ The setup for great apes was protected by Plexiglas boxes; chimpanzee and gorilla setups were fixed in the enclosure, the one used with the orangutans was installed on a trolley allowing the experimenter to move between the enclosures (Figure S4).

A daily testing session started when one individual approached and pressed the screen, then, participants saw approximately ten testing trials. As we did not want to induce any bias in the participants’ choices, there was no sound feedback or food reward directly after participants made a choice. Thus, a single test trial could broadly be divided into three parts: participants (*i*) watched the clip, (*ii*) chose one actor on the last frame, and (*iii*) pressed on an image (a human hand holding a fruit for the great ape participants, and a trophy cup for the humans) to get a positive sound and a food reward, for the great apes only (Figure S1). If they pressed outside the image, a negative sound was emitted, and they had to try again until they succeeded. Once a participant left the testing area, the experimenter ended the test. If the participant returned later, the session resumed where it had stopped.

#### Stimuli

Stimuli were short, soundless, colored natural video clips (see other supplementary materials), presented either in their original or mirrored version, to counterbalance the side of the agent across trials.

In the *Animate>Animate* condition, the two actors belonged to the same species but could have different perceptual features. The nature of the interaction (agonistic, play, affiliative or cooperative) varied as well, but the different event categories could be recognized by all participants, as affiliation and aggression share similar characteristics across species.^48^ The clips were sorted into five blocks based on the species of the actors: chimpanzees, gorillas, humans, non-primates (e.g., dogs, elephants, birds …) or orangutans.

All participants started with clips of conspecifics, as we expected it would be easier for them to detect goals and thus to understand the event. The remaining four blocks were randomised. Each block consisted of 15 events, except for the human block that only had 14 clips as one might have upset younger children (chimpanzee mean duration of the video clips (±SD): 6.4s ±2.5; gorilla: 4.7s ±2.1; human: 4.3s ±0.9; non-primates: 6.0s ±1.8 and orangutan: 5.2s ±1.6).

Before moving to the other conditions, great apes went through a ten trials session of *Inanimate>Inanimate* events, to ensure that they were able to generalise the task to inanimate actors. As the accuracy rate was approximately 85% (mean number of touches necessary to achieve a valid choice: 1.9 ± 2.9; median = 1) we decided that additional training was unnecessary.

We presented 38 *Inanimate>Inanimate* events (4.0s ±1.7) depicting different objects, such as balls, cars, trees, in different types of interaction (e.g., colliding, falling on). In the *Inanimate>Animate* condition we presented 15 clips (3.7s ±1.1) of various objects acting on a human patient, such as a ball smashing into the face of a person. Finally, in the *Animate>Inanimate* condition we presented 25 clips (6.6s ±1.9), where the animate actors, from the same species as in the *Animate>Animate* condition, manipulated various objects (e.g., a cow playing with a ball, an orangutan manipulating with Lego blocks).

In addition to the clips presenting interactions, we also showed control video clips that differed only insofar as there was no interaction between the two actors (*Animate/Animate*: *N* = 25, mean (±SD): 4.3s ±1.4; *Animate/Inanimate*: *N* = 7, mean (±SD): 3.7s ±1.4; *Inanimate/Inanimate*: *N* = 5, mean (±SD): 3.0s ±0.7), and allowed us to test the influence of the interaction on the choice.

The same great apes participated in all four conditions, whereas human adults and children participants were split, half of them were exclusively tested in the *Animate>Animate* condition, while the remaining half were tested in the three other conditions, to maximise our chance of recruiting participants from the zoo visitors. Great apes always started with the *Animate>Animate* condition to maximise their interest for the study and all species, except children, saw *Inanimate>Inanimate* events, followed by *Inanimate>Animate* events and finished by the *Animate>Inanimate* condition. These blocks were not randomised because we wanted to avoid any bias for the animate actor. Children saw the different conditions in a random order. Great ape and adult participants were tested on all the clips, while, for constraints of time and attention span (as participants were recruited amongst the zoo visitors), the children were tested with subsets (Table S7).

#### Training of the apes

Only great ape participants received training before the experiment. The training consisted in watching short, soundless, and colored natural video clips, of a single unknown conspecific actor in his environment either at rest or moving. For each great ape species, we used a set of 92 clips (chimpanzee: mean (±SD) duration 4.9s ±0.6; gorilla: 3.8s ±1.4; and orangutan: 4.1s ±1.3). The clips were short to maximise the attention and interest of the subjects.

Our training was designed as a three-stage process. Each stage began with the participants watching a video clip, followed by a still image of the last frame remaining on screen. The content of the still image varied according to the training phase. In the first phase, the silhouette of the actor appeared on a white background. In the second, the background was blurred, while it remained unchanged in the third phase (Figure S5). The task difficulty also gradually increased throughout the training. In the first phase, participants were allowed to touch the screen as many times as needed to reach the target (the area of the actor), but the trial was considered as failed if more than one touch was necessary. To pass this first training phase, participants had to obtain an 80% success rate (or more) for two consecutive sessions of ten trials a day. The second training phase was quite similar, except the criteria of success was three consecutive sessions at 80% success rate. Finally, in the last phase, participants only had one chance to correctly touch the actor, if not the trial was a failure, and they moved on to the next one. The criterion of success was the same as for the second training phase. During the entire training, we used positive reinforcement based on sound feedback and food reward.

#### Testing procedure for the great apes

A daily testing session, for the great apes, always started when one individual approached and pressed a green screen (followed by a food reward). Then, five warm-up trials (random images) were presented to help the participants to focus on work, followed by three training trials (phase three). Then, participants saw approximately ten testing trials before finishing with five cool-off trials (random images) and a red screen with three pieces of grapes (Figure S1). Human participants did not go through the warm-up and training trials and directly started with the test trials. They saw all clips in a row, with a 5-min break halfway through the session if needed.

### Quantification and statistical analysis

Statistical analyses were conducted in R (R Core Team, 2021, version 4.0.5) using Bayesian generalised linear models through the brms package^65^^,^^66^^,^^67^ interface to Stan.^62^ We chose a Bayesian framework because it is less dependent on sample size and allows richer assessment of uncertainty in parameter estimates.

Log-linear models were used to rule out that a choice was conditionally independent of the interaction between the actors, i.e., exchangeable across control and test videos. To compute this, we created a contingency table of summed choices and fitted Bayesian regression with a Poisson distribution (normally distributed priors: μ = 0, σ = 1.5). In one model the type of test (control or test) was included and in the second one it was not (see key resources table for formal model definitions). We compared the expected log pointwise predictive density (elpd) under leave-one-out cross validation to select the best model. Higher elpd indicates higher out-of-sample predictive fit, thus a better model.

Bayesian Bernoulli models, with a logit link, were fitted (*i*) to model the agent choice according to the species of the participant and across conditions but also their interactions and (*ii*) model the agent choice regarding the species of the participant and the type of interaction in the *Animate>Animate* condition, as well as the interaction between the two. Normally distributed priors (μ = 0, σ = 1.5) were used for the intercept and all population-level predictors (which correspond to a relatively flat distribution on the logit scale for each predictor), while exponentially distributed priors (λ = 1) were used for the standard deviation of group-level effects. The outcome variable was the choice made and the predictors of interest were the species of the participant and the condition or the content of the event, as well as their interactions.

To control for potential confounding factors, both models included as fixed predictors the different features of the clip (e.g., the side of the choice, the side of the agent, the relative size difference between agent and patient, see Table S1 for details and coding of these features). These same predictors were also included as random slopes by participant and clip (stimulus). Formal model definitions are presented in the Supplementary Text.

Given the large number of potential confounds we performed variable selection using leave-one-out cross validation (*cv_varsel* function of the *projpred* package^68^ with optimised parallelization) to trim the model down to only those covariates that are needed to retain the same out-of-sample predictive performance. From the 14 predictors only 8 were kept and used for all models (see Figure S2).

All models were checked for convergence based on trace plots, R-hat values and ESS diagnostics. We further controlled for the fit of the model using the *pp_check* function, which compared the observed data to the data simulated from the model’s posterior predictive distribution (see Figure S6). We also conducted sensitivity checks by fitting the models with different priors (Normal(0,1.5); Normal(0,0.5) and Horseshoe(1)) and we did not get any noticeable differences, suggesting that the model learned parameters from the data and not the prior despite the relatively small sample size. Finally, we controlled for overly influential data points with Pareto *k* estimates.^69^^,^^70^ All Pareto *k* were within the commonly accepted range (*k* < 0.5), suggesting that there were no overly influential data points and that our *elpd* estimates were reliable.

For each parameter of interest, we reported its posterior estimate mean and standard error, as well as the proportion of the posterior estimates above a 0.5 choice (main text) and the 90% credible intervals, which we considered as the meaningful threshold (Table S2). We visualised this information with density plots of the posterior distribution. In these figures, in addition to the 90% CI we also reported the 30% and 60% CIs.

## Supplemental information

Document S1. Figures S1‒S6 and Tables S1‒S7
