# Supplementary material for: A universal preference for animate agents in hominids
Source: iScience. 2024 May 16;27(6):109996. doi: 10.1016/j.isci.2024.109996 (PMC11177197; doi:10.1016/j.isci.2024.109996)
Supplement: Document S1. Figures S1‒S6 and Tables S1‒S7 [file mmc1.pdf]

iScience, Volume *27*

Supplemental information

A universal preference for animate agents in hominids

Sarah Brocard, Vanessa A.D. Wilson, Chloé Berton, Klaus Zuberbühler, and Balthasar Bickel

This PDF file includes:

Figures S1 to S6
Tables S1 to S7

Other Supplementary Materials for this manuscript include the following:

Dataset S1
Scripts S1 to S5
R Data Files S1 to S5
Formal definition of the statistical models
Stimuli

They are available at: https://osf.io/5vrc7/?view_only=3519ecd7e366403d9f8e9e52cb17eb71

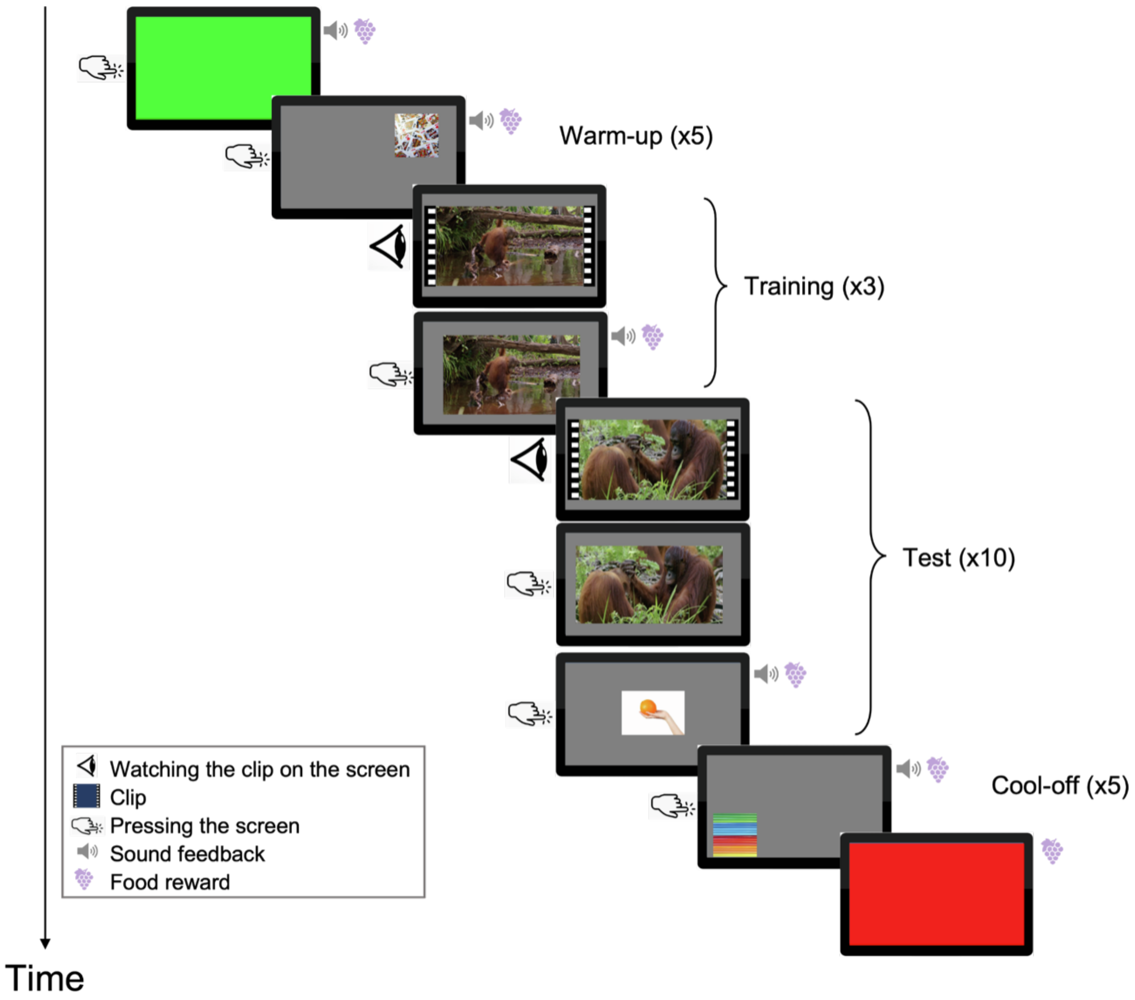

Figure S1: General schematic procedure for a testing session (related to STAR Methods). A session started with a green screen that needed to be pressed to get sound feedback and food reward (for great apes only). Then, great ape participants went through five trials of random images of 380 x 380 pixels and three trials of the third phase of the training. Approximately ten test trials followed. The session ended with five new trials of random images and the red screen. The clips were played full screen, the still images were displayed at 1920 x 920 pixels and the fruit and cup images at 600 x 600 pixels. The human participants started with a green screen and directly went to the test trials.

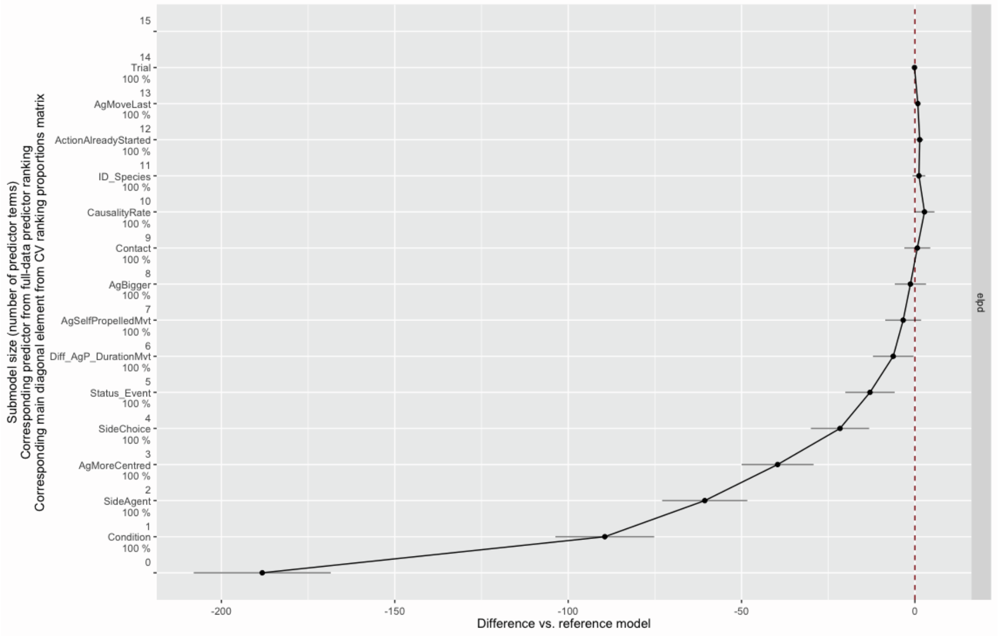

Figure S2: Results of the variables selection (related to STAR Methods).

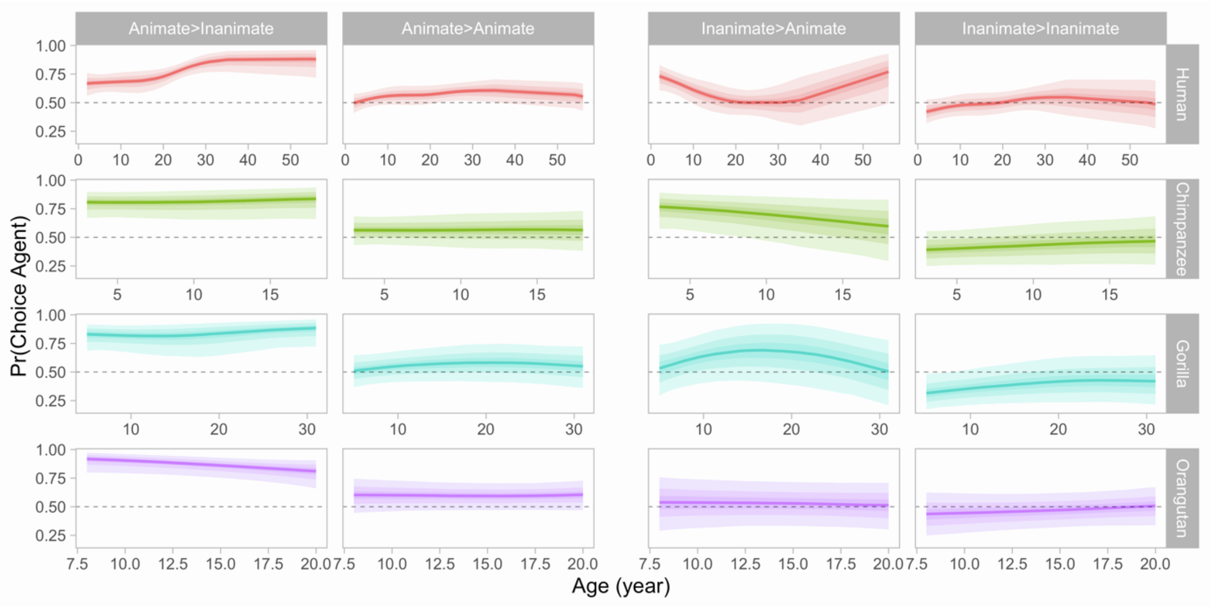

Figure S3: Posterior probability of the agent choice for each species across conditions and age, extracted from the gam Bayesian model (related to Fig. 1A). Ages were centred for each participant in the model. The dashed line corresponds to a random choice (50%). The thick lines represent the mean of the estimates and the three shade levels represent the 30, 60 and 90% credible intervals. Twenty human adults, fifty human children, four chimpanzees, four gorillas and five orangutans were tested for each condition, but one adult was removed from the AN>AN condition. Human adults and children were merged in this analysis. Twenty-five clips were presented in the AN>IN condition, 41 in the IN>IN, 74 in the AN>AN and 15 in the IN>AN condition. AN stands for animate, IN for inanimate and “>” for “acting on”.

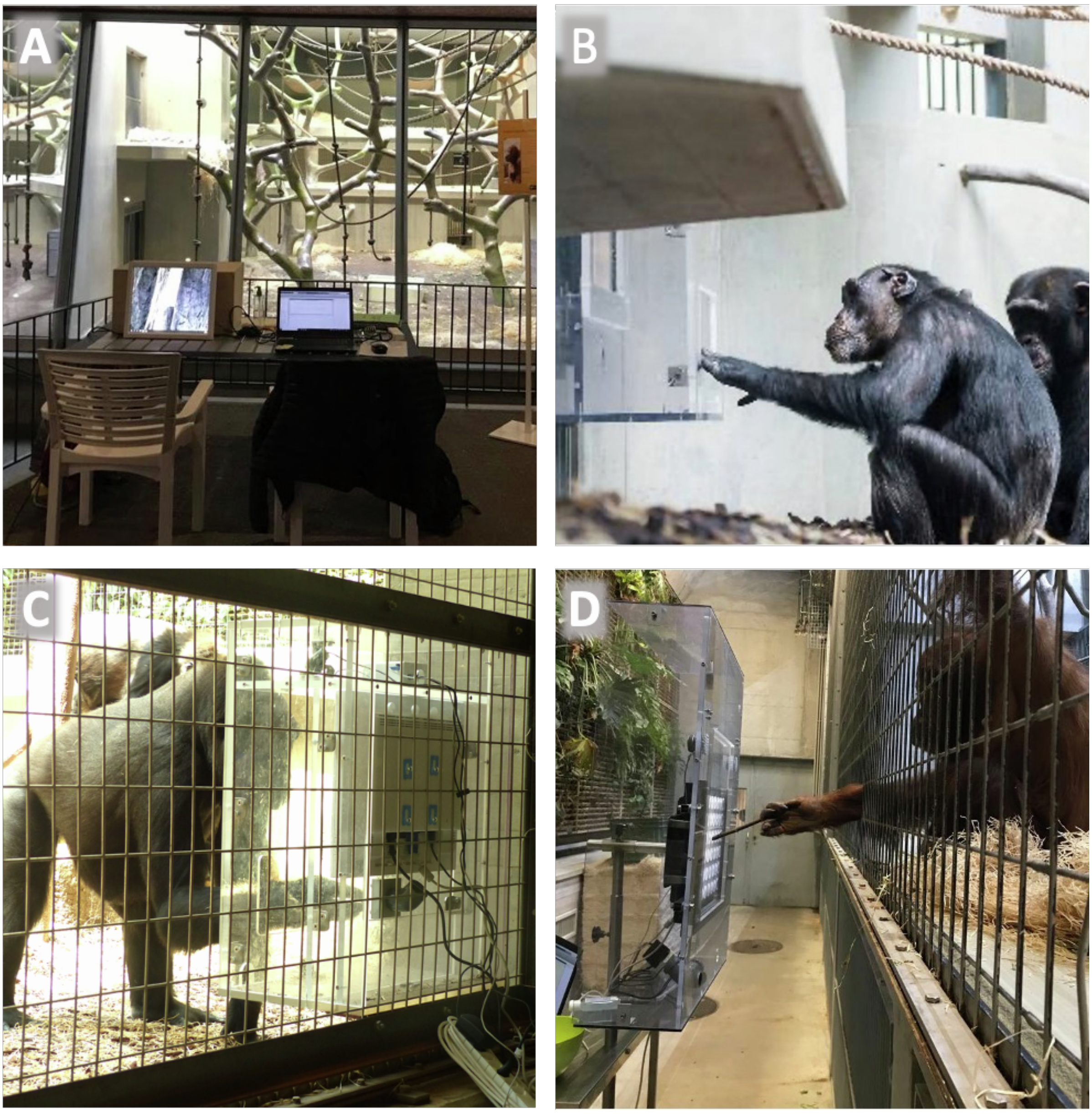

Figure S4: Different setups used in the study (related to STAR Methods). (A) setup used for the humans, (B) fixed setup in the chimpanzees' enclosure, (C) fixed setup in the gorillas' enclosure and (D) movable setup used to test the orangutans. Photo credits: (A-C-D) S. Brocard and (B) Zoo Basel.

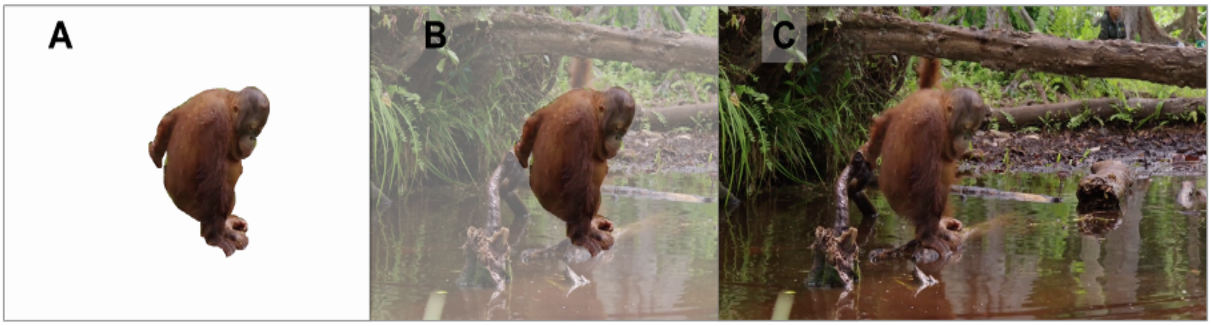

Figure S5: Example of a still image across three-stage training (related to STAR Methods). (A) phase 1 with the white background; (B) phase 2 with the blurred background and (C) phase 3 with the natural one. Photo credit: Orangutan Jungle School, Season #1, NHNZ Worldwide.

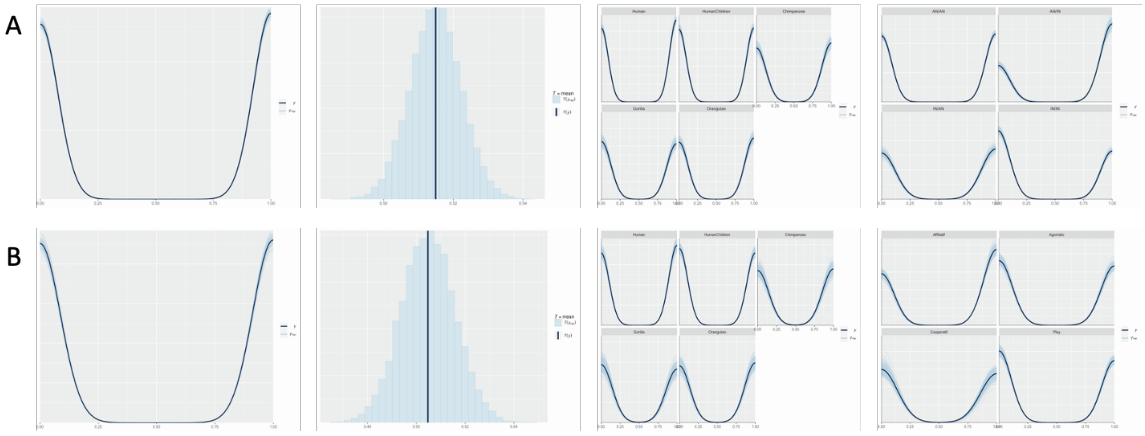

Figure S6: Posterior predictive checks for (A) the conditions model and (B) content model (related to STAR Methods). The first panels compare the posterior predictive distribution (y_{rep}) with the observed data (y). The second is predictive distribution of the mean compared to the observed mean. The third and fourth are divided by species and conditions (for A) and content of the event (for B). All posterior predictions capture the data very well.

Table S1: Details of the variable used and coding (related to STAR Methods).

Variable	Variable Name	Measurement Unit	Description
AgMoreCentred	Agent is more centred	Factor	The agent is closer to the centre of the screen than the patient is, on the still image. 3 levels, 0: the patient is more centred; 1: the agent is more centred; 2: both actors are as far from th centre.
AgSelfPropelledMvt	Agent is self-propelled movement	Factor	The agent is self-propelled. 2 levels, 0: no; 1: yes.
c_Years	Centred participant age (year)	Numeric	Age centred for each participant.
ChoiceAg	Choice is the agent	Integer	The choice of the participant was the agent. 2 levels, 0: no; 1: yes.
clipName_NoFLIP	Clip name	Factor	Name of the clip, original and flip versions ar considered alike. 155 levels.
Condition	Condition	Factor	Condition in which the event belongs to. 4 levels. AN>AN (animate agent acts on animate patient); AN>IN (animate agent>inanimate patient); IN>AN and IN>IN.
Diff_AgP_DurationMvt	Difference agent-patient for duration of movements	Numeric	Difference (agent - patient) of total time moving in the event. If negative the patient is moving more than the agent and if positive it is the agent that moves longer than the patient
Grouped_Content_AnA	Content of the stimulus in the AN>AN conditio	Factor	Content of the events in the AN>AN conditio only. 4 levels, affiliative; agonistic; cooperative; play.
ID	Participant ID name/number	Factor	Name of the great apes or ID number assigned to the participant (starting from H1 for adults and s01 for children; assigning in sequential order).
Species	Participant specie	Factor	Species of the participant (chimpanzee, gorilla, human adult, human children and

			orangutan).
Species_Combine	Participant specie (humans combined)	Factor	Species of the participant (chimpanzee, gorilla, human and orangutan). Human adults and children were combined.
SideAgent	Side of the agent	Factor	Side of the agent on the still image. 3 levels, AL (agent on left-hand side); AR (agent on th right-hand side); ATop (agent on top).
SideChoice	Side of the choic	Factor	On the still image the side of the actor the participant pressed on. 2 levels, left; right.
Status_Event	Status of the even	Factor	Status of the event. 2 levels, N: the event is still ongoing at the end of the video clip and thus on the still image; Y: the event is finishe at the end of the video clip.

Table S2: Detailed posterior estimates of the Bayesian Bernoulli regressions for each species in both models (related to Fig. 1).

	Condition model					*Content model*				
Species	Condition	Mean	SD	Credible Interval 5%	Credible Interval 95%	Content	Mean	SD	Credible Interval 5%	Credible Interval 95%
Human adults	AN>IN	0.790	0.049	0.706	0.864	Agonistic	0.514	0.056	0.422	0.606
Human children		0.693	0.057	0.595	0.783		0.576	0.055	0.483	0.665
Chimpanzees		0.812	0.085	0.651	0.924		0.498	0.097	0.335	0.656
Gorillas		0.831	0.078	0.683	0.932		0.498	0.099	0.334	0.660
Orangutan		0.852	0.070	0.719	0.941		0.643	0.095	0.476	0.789
Human adults	AN>AN	0.577	0.043	0.505	0.646	Play	0.522	0.046	0.447	0.596
Human children		0.535	0.043	0.463	0.606		0.518	0.045	0.444	0.592
Chimpanzees		0.568	0.067	0.455	0.677		0.552	0.081	0.416	0.682
Gorillas		0.530	0.067	0.420	0.640		0.485	0.083	0.347	0.622
Orangutan		0.600	0.063	0.493	0.700		0.502	0.088	0.357	0.647
Human adults	IN>AN	0.543	0.085	0.401	0.682	Affiliative	0.674	0.050	0.588	0.753
Human children		0.692	0.074	0.562	0.805		0.494	0.057	0.401	0.587
Chimpanzees		0.717	0.128	0.481	0.896		0.605	0.089	0.451	0.746
Gorillas		0.542	0.151	0.285	0.784		0.595	0.093	0.436	0.743
Orangutan		0.556	0.149	0.303	0.794		0.656	0.091	0.497	0.797
Human adults	IN>IN	0.513	0.062	0.412	0.615	Cooperative	0.650	0.068	0.534	0.757
Human children		0.456	0.061	0.355	0.557		0.435	0.075	0.314	0.562
Chimpanzees		0.421	0.100	0.261	0.591		0.513	0.132	0.293	0.725
Gorillas		0.363	0.096	0.215	0.531		0.571	0.131	0.348	0.776
Orangutan		0.475	0.100	0.314	0.641		0.486	0.142	0.256	0.724

Note. Agent choice across conditions and across types of interaction in the *Animate*>*Animate* condition. We reported the mean posterior estimates and estimated errors of fixed parameters of interest. All posterior estimates are given in Tab. S3 and S4. AN stands for animate, IN for inanimate and “>” for “acting on”.

Table S3: Detailed posterior estimates of the Bayesian Bernoulli regression modelling the agent choice across species and conditions (related to Fig. 1A).

Parameter	Mean	Est.Error	l-90% CI	u-90% CI	Post.Prob
Condition (= AN>AN)	-0.624	0.303	-1.126	-0.124	0.98
Condition (= AN>IN)	0.416	0.381	-0.205	1.045	
Condition (= IN>AN)	-0.757	0.402	-1.419	-0.088	0.97
Condition (= IN>IN)	-0.881	0.318	-1.395	-0.351	1
Species (= Human children)	-0.169	0.139	-0.398	0.059	
Species (= Chimpanzee)	-0.035	0.252	-0.450	0.380	
Species (= Gorilla)	-0.189	0.253	-0.605	0.232	
Species (= Orangutan)	0.100	0.238	-0.295	0.489	
SideChoice (= Right)	-0.371	0.059	-0.469	-0.273	1
SideAgent (= Right)	0.154	0.082	0.020	0.288	0.97
SideAgent (= Top)	-0.814	0.253	-1.231	-0.402	1
Status_Event (= Y)	0.387	0.139	0.157	0.613	
AgSelfPropelledMvt	0.335	0.252	-0.082	0.750	
AgMoreCentred (= 1)	0.473	0.173	0.188	0.756	1
AgMoreCentred (= 2)	0.418	0.154	0.165	0.668	1
Diff_AgP_DurationMvt	0.064	0.029	0.017	0.111	0.99
Condition x Species (= AN>IN x Human children)	-0.352	0.337	-0.913	0.197	
Condition x Species (= IN>AN x Human children)	0.822	0.409	0.162	1.494	0.98
Condition x Species (= IN>IN x Human children)	-0.066	0.265	-0.498	0.371	
Condition x Species (= AN>IN x Chimpanzee)	0.242	0.584	-0.716	1.196	
Condition x Species (= IN>AN x Chimpanzee)	0.877	0.664	-0.215	1.967	
Condition x Species (= IN>IN x Chimpanzee)	-0.353	0.413	-1.031	0.319	
Condition x Species (= AN>IN x Gorilla)	0.532	0.591	-0.433	1.497	
Condition x Species (= IN>AN x Gorilla)	0.194	0.671	-0.905	1.284	
Condition x Species (= IN>IN x Gorilla)	-0.452	0.417	-1.140	0.226	
Condition x Species (= AN>IN x Orangutan)	0.403	0.570	-0.527	1.34	
Condition x Species (= IN>AN x Orangutan)	-0.030	0.648	-1.093	1.037	
Condition x Species (= IN>IN x Orangutan)	-0.258	0.400	-0.914	0.398	

Note. We reported the mean posterior estimates and estimated errors of all fixed parameters. We additionally included the posterior probability that a given coefficient is above or below 0 on the logit scale, corresponding to a .5 probability of agent choice. In the model all Pareto *k* estimates were good (k < 0.5), suggesting that results were not driven by overly influential datapoints and that our *elpd* estimates are reliable. AN stands for animate, IN for inanimate and “>” for “acting on”.

Table S4: Detailed posterior estimates of the Bayesian Bernoulli regression modelling the agent choice across species and the type of interaction (related to Fig. 1B).

Parameter	Mean	Est.Error	l-90% CI	u-90% CI	Post.Prob
Species (= Human)	0.064	0.234	-0.317	0.451	
Species (= Human children)	-0.694	0.235	-1.080	-0.310	1
Species (= Chimpanzee)	-0.229	0.386	-0.856	0.404	
Species (= Gorilla)	-0.272	0.397	-0.919	0.373	
Species (= Orangutan)	0.002	0.412	-0.681	0.680	
Content (= Agonistic)	-0.678	0.227	-1.056	-0.308	1
Content (= Cooperative)	-0.100	0.283	-0.565	0.363	
Content (= Play)	-0.647	0.214	-1.004	-0.297	1
SideChoice (= Right)	-0.021	0.081	-0.151	0.112	
SideAgent (= Right)	0.035	0.115	-0.154	0.223	
Status_Event (= Y)	0.246	0.148	0.004	0.489	0.95
AgMoreCentred (= 1)	0.456	0.181	0.158	0.751	0.99
AgMoreCentred (= 2)	0.315	0.147	0.074	0.559	0.98
Diff_AgP_DurationMvt	0.106	0.036	0.048	0.166	1
Species x Content (= Human children x Agonistic)	1.011	0.248	0.607	1.419	1
Species x Content (= Chimpanzee x Agonistic)	0.226	0.439	-0.508	0.929	
Species x Content (= Gorilla x Agonistic)	0.271	0.456	-0.491	1.012	
Species x Content (= Orangutan x Agonistic)	0.620	0.473	-0.159	1.383	
Species x Content (= Human children x Cooperative)	-0.143	0.323	-0.669	0.385	
Species x Content (= Chimpanzee x Cooperative)	-0.285	0.565	-1.226	0.645	
Species x Content (= Gorilla x Cooperative)	0.008	0.583	-0.943	0.965	
Species x Content (= Orangutan x Cooperative)	-0.631	0.616	-1.649	0.364	
Species x Content (= Human children x Play)	0.741	0.230	0.365	1.117	1
Species x Content (= Chimpanzee x Play)	0.421	0.406	-0.247	1.088	
Species x Content (= Gorilla x Play)	0.186	0.426	-0.512	0.882	
Species x Content (= Orangutan x Play)	-0.016	0.444	-0.754	0.705	

Note. Clips from the Animate>Animate condition (“>” stands for “acting on”). We reported the mean posterior estimates and estimated errors of all fixed parameters. We additionally included the posterior probability that a given coefficient is above or below 0 on the logit scale, corresponding to a .5 probability

of agent choice. In the model all Pareto k estimates were good ($k < 0.5$), suggesting that results were not driven by overly influential datapoints and that our *elpd* estimates are reliable.

Table S5: Detailed posterior estimates of the gam Bayesian regression modelling the agent choice across age, species, and conditions (related to Fig. 1A).

Parameter	Mean	Est.Error	l-90% CI	u-90% CI	Post.Prob
Condition (= AN>AN)	-0.660	0.289	-1.136	-0.187	0.99
Condition (= AN>IN)	0.163	0.312	-0.348	0.673	
Condition (= IN>AN)	-0.575	0.301	-1.071	-0.081	0.97
Condition (= IN>IN)	-0.949	0.281	-1.409	-0.490	1
Species_Combine (= Chimpanzee)	-0.025	0.347	-0.591	0.552	
Species_Combine (= Gorilla)	-0.127	0.283	-0.587	0.340	
Species_Combine (= Orangutan)	0.179	0.349	-0.372	0.760	
SideChoice (= Right)	-0.368	0.058	-0.464	-0.272	1
SideAgent (= Right)	0.151	0.082	0.018	0.286	0.97
SideAgent (= Top)	-0.776	0.243	-1.179	-0.381	1
Status_Event (= Y)	0.370	0.133	0.154	0.587	1
AgSelfPropelledMvt	0.338	0.243	-0.058	0.744	
AgMoreCentred (= 1)	0.462	0.165	0.189	0.734	1
AgMoreCentred (= 2)	0.438	0.141	0.205	0.672	1
Diff_AgP_DurationMvt	0.058	0.027	0.013	0.103	0.99
Condition x Species_Combine (= AN>IN x Chimpanzee)	0.451	0.452	-0.274	1.188	
Condition x Species_Combine (= IN>AN x Chimpanzee)	0.666	0.526	-0.196	1.533	
Condition x Species_Combine (= IN>IN x Chimpanzee)	-0.324	0.395	-0.977	0.319	
Condition x Species_Combine (= AN>IN x Gorilla)	0.699	0.401	0.048	1.363	0.96
Condition x Species_Combine (= IN>AN x Gorilla)	0.082	0.477	-0.696	0.858	
Condition x Species_Combine (= IN>IN x Gorilla)	-0.448	0.344	-1.017	0.110	
Condition x Species_Combine (= AN>IN x Orangutan)	0.673	0.496	-0.133	1.471	
Condition x Species_Combine (= IN>AN x Orangutan)	-0.429	0.505	-1.272	0.387	
Condition x Species_Combine (= IN>IN x Orangutan)	-0.268	0.394	-0.927	0.365	
sc_Years	0.124	1.349	-2.104	2.352	NA
sc_Years x Condition (= AN>AN)	0.170	1.366	-2.060	2.414	NA
sc_Years x Condition (= AN>IN)	-0.142	1.456	-2.529	2.259	NA
sc_Years x Condition (= IN>AN)	-0.166	1.449	-2.554	2.219	NA

sc_Years x Condition (= IN>IN)	0.258	1.398	-2.055	2.543	NA
sc_Years x Species_Combine (=Human)	0.153	1.365	-2.105	2.382	NA
sc_Years x Species_Combine (= Chimpanzee)	-0.051	1.466	-2.464	2.358	NA
sc_Years x Species_Combine (= Gorilla)	0.081	1.461	-2.327	2.497	NA
sc_Years x Species_Combine (= Orangutan)	-0.048	1.470	-2.488	2.384	NA
sc_Years x Condition x Species_Combine (= AN>AN x Human)	0.146	1.387	-2.168	2.435	NA
sc_Years x Condition x Species_Combine (= AN>IN x Human)	0.105	1.450	-2.293	2.448	NA
sc_Years x Condition x Species_Combine (= IN>AN x Human)	-0.055	1.458	-2.442	2.312	NA
sc_Years x Condition x Species_Combine (= IN>IN x Human)	-0.060	1.384	-2.326	2.217	NA
sc_Years x Condition x Species_Combine (= AN>AN x Chimpanzee)	-0.008	1.440	-2.376	2.35	NA
sc_Years x Condition x Species_Combine (= AN>IN x Chimpanzee)	0.028	1.488	-2.431	2.492	NA
sc_Years x Condition x Species_Combine (= IN>AN x Chimpanzee)	-0.221	1.463	-2.615	2.169	NA
sc_Years x Condition x Species_Combine (= IN>IN x Chimpanzee)	0.123	1.514	-2.361	2.606	NA
sc_Years x Condition x Species_Combine (= AN>AN x Gorilla)	-0.004	1.447	-2.379	2.371	NA
sc_Years x Condition x Species_Combine (= AN>IN x Gorilla)	-0.010	1.471	-2.426	2.409	NA
sc_Years x Condition x Species_Combine (= IN>AN x Gorilla)	0.055	1.462	-2.352	2.461	NA
sc_Years x Condition x Species_Combine (= IN>IN x Gorilla)	0.039	1.445	-2.336	2.414	NA
sc_Years x Condition x Species_Combine (= AN>AN x Orangutan)	0.024	1.493	-2.395	2.456	NA
sc_Years x Condition x Species_Combine (= AN>IN x Orangutan)	-0.302	1.476	-2.738	2.115	NA
sc_Years x Condition x Species_Combine (= IN>AN x Orangutan)	0.061	1.494	-2.369	2.523	NA
sc_Years x Condition x Species_Combine (= IN>IN x Orangutan)	0.152	1.469	-2.273	2.597	NA

Note. We reported the mean posterior estimates and estimated errors of all fixed parameters. We additionally included the posterior probability that a given coefficient is above or below 0 on the logit scale, corresponding to a .5 probability of agent choice, we couldn't test the hypotheses for the splines. In

the model all Pareto k estimates were good ($k < 0.5$), suggesting that results were not driven by overly influential datapoints and that our *elpd* estimates are reliable. AN stands for animate, IN for inanimate and “>” for “acting on”.

Table S6: Detailed information of great apes who participated in the study (related to STAR Methods).

Species	Name	Sex	Age
Chimpanzee	Kume	M	18
Chimpanzee	*Obaye*	M	4
Chimpanzee	*Obuasi*	F	4
Chimpanzee	*Ponima*	F	3
Gorilla	Adira	F	14
Gorilla	Joas	F	31
Gorilla	*Makala*	F	5
Gorilla	*Mobali*	M	5
Orangutan	Bagus	M	19
Orangutan	Budi	M	17
Orangutan	*Ketawa*	F	8
Orangutan	Kila	F	21
Orangutan	Maia	F	14
Orangutan	Vendel	M	21

Note. Names in italic are the immature individuals. F: female; M: male.

Table S7: Number of clips per condition (related to STAR Methods).

Conditions	Adults	Children	Chimpanzee	Gorillas	Orangutans
AN>AN	74	25	74	74	74
IN>IN	41	9	41	41	41
IN>AN	15	4	15	15	15
AN>IN	25	7	25	25	25
**Total**	**155**	**45**	**155**	**155**	**155**

Note. Children saw only subsets of all the clips for constraints of time and attention span (as participants were recruited amongst the zoo visitors). AN stands for animate and IN for inanimate, where “>” stands for “acting on”.
